# Review of Laser Raman Spectroscopy for Surgical Breast Cancer Detection: Stochastic Backpropagation Neural Networks

**DOI:** 10.3390/s20216260

**Published:** 2020-11-02

**Authors:** Ragini Kothari, Yuman Fong, Michael C. Storrie-Lombardi

**Affiliations:** 1 Department of Surgery, City of Hope, 1500 E. Duarte Rd., Duarte, CA 91010, USA; yfong@coh.org; 2 Kinohi Institute, Inc., Santa Barbara, CA 93109, USA; mike@kinohi.org; 3 Department of Physics, Harvey Mudd College, Claremont, CA 91711, USA

**Keywords:** Raman spectroscopy, breast cancer, neural networks, multivariate statistics

## Abstract

Laser Raman spectroscopy (LRS) is a highly specific biomolecular technique which has been shown to have the ability to distinguish malignant and normal breast tissue. This paper discusses significant advancements in the use of LRS in surgical breast cancer diagnosis, with an emphasis on statistical and machine learning strategies employed for precise, transparent and real-time analysis of Raman spectra. When combined with a variety of “machine learning” techniques LRS has been increasingly employed in oncogenic diagnostics. This paper proposes that the majority of these algorithms fail to provide the two most critical pieces of information required by the practicing surgeon: a probability that the classification of a tissue is correct, and, more importantly, the expected error in that probability. Stochastic backpropagation artificial neural networks inherently provide both pieces of information for each and every tissue site examined by LRS. If the networks are trained using both human experts and an unsupervised classification algorithm as gold standards, rapid progress can be made understanding what additional contextual data is needed to improve network classification performance. Our patients expect us to not simply have an opinion about their tumor, but to know how certain we are that we are correct. Stochastic networks can provide that information.

## 1. Introduction

Breast cancer is the second most commonly diagnosed cancer among American women after skin cancer, with approximately 1 in 8 women (12.8%) receiving a diagnosis of invasive breast cancer within their lifetime [1]. Early detection of tumors has become possible due to technological advancement of screening techniques as well as public education and awareness [2]. The standard of care for early stage invasive breast cancer includes breast irradiation and breast conserving surgery (BCS), during which the surgeon attempts to excise all tumors with a negative margin, that is margins of the resected tissue have no evidence of tumor [2,3]. Current gold standard definition of a negative surgical margin is defined as no malignant cells at the surface of resected tissue specimen [4]. Large studies have been conducted to compare outcomes between BCS and traditional mastectomy procedures, showing equivalent overall survival rates [5]. Mastectomy procedures include undesirable cosmetic outcomes for the patient, increased psychological burden and increased wound infection risk [5]. Unfortunately, the biggest risk with BCS is the chance of local reoccurrence (LR) with 15% to 35% of patients who opt for BCS requiring a second surgery to obtain negative margins [4,5]. Current standard of care after BCS includes a pathological investigation of tumor margins using hematoxylin and eosin (H&E) stains [4]. During this process the excised tissue is fixed, processed, sectioned and stained with H&E and studied under a microscope. This process is time and labor intensive, with results only becoming available days after initial surgery. If the surgical pathologist finds positive or close to positive margins, the patient has to undergo a second surgery. Hence, the accurate detection of tumor margins during surgery has become an area of intense investigation and technology development. Some intraoperative modalities that are under investigation to tackle this problem include radiofrequency spectroscopy [6], bioimpedance spectroscopy [7], photoacoustic tomography [8], spatial frequency domain imaging (SFDI) [9], fluorescence imaging [10,11], elastic scattering spectroscopy [12], microscopy with ultraviolet surface excitation (MUSE) [13], light-sheet microscopy [14], nonlinear microscopy [15], optical coherence tomography [16] and laser Raman spectroscopy (LRS) [17]. Maloney et al. review all of these intraoperative techniques as well as pre-operative imaging technologies for margin detection during BCS [4]. Their recommendation is a hybrid system of imaging modalities and optical scanning techniques to achieve desired sensitivity and specificity required for margin detection. In recent years, many investigators have pointed out the advantages of using a highly specific biomolecular probe, laser Raman spectroscopy (LRS), to distinguish breast cancer tumors from healthy breast tissue and benign masses [17,18,19,20,21].

**Figure 1:**
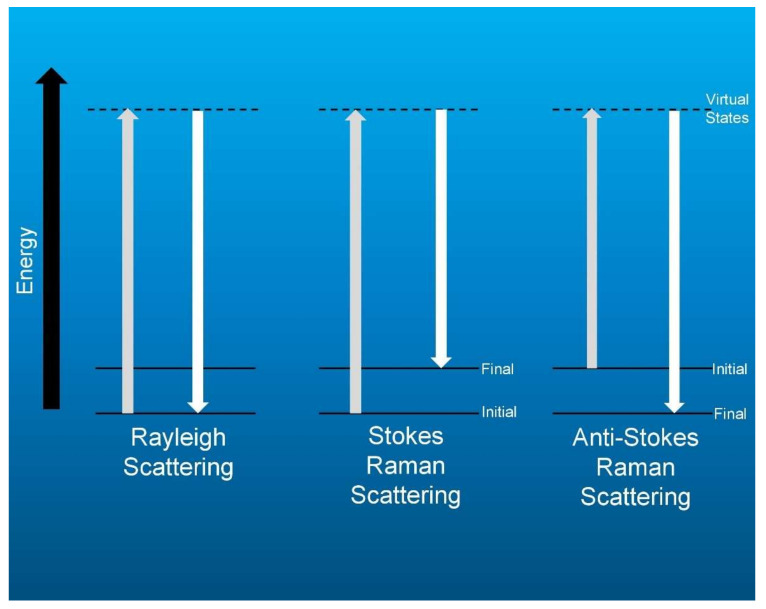
Energy level diagram of the Raman effect.

This article focuses on LRS as a real-time tool for breast tumor detection during surgery. LRS is a non-destructive and label free technique that harnesses the biochemical specificity of the Raman effect [22]. A tiny fraction of incident light on an object is absorbed by its molecules which are set into vibrational motion and the subsequent frequency change of the scattered light is equal to the vibrational frequency of the these molecules [22]. The metric for Raman spectroscopy is known as the Stokes shift, named after Irish physicist George Gabriel Stokes. In the mid-19th century, Stokes noted that while most light incident on matter was either absorbed or reflected back at the same wavelength as the illuminating light (Rayleigh scatter) some light came back from the interaction at a longer wavelength; he coined the term “fluorescence” for this lower energy light [23]. This shift in wavelength to lower energy state occurs in both fluorescence and Raman scattering and was ultimately referred to as a Stokes shift in his honor. The origin of the Stokes shift is commonly represented in a Perrin-Jablonski diagram (Figure 1).

The energy transfer occurs between the incident photons and the electrons of the target atoms. If the electrons are in the ground state a Stokes shift occurs with a loss of photon energy. If the electrons are in an excited state, the photons can gain energy in what is termed an anti-Stokes shift. This scattered light is collected by a spectrometer, creating a spectrum, which showcases a series of peaks, also referred to as bands, corresponding to the characteristic vibrational frequencies of the scattering molecules [22]. This creates a unique biomolecular ‘fingerprint’ of the sample. When applied to cancer diagnostics, this unique fingerprint can be a direct indicator of the inherent biochemical differences between malignant and healthy tissue. Such biochemical specificity obtained from the surgical field can be instrumental in helping a surgeon achieve negative margins during BCS.

This paper discusses the most significant research advancements in the use of LRS as a real-time in vivo tool for surgical breast cancer detection, with an emphasis on the statistical and machine learning methods employed for spectral classification. The choice and implementation of machine learning diagnostic algorithms is critical for precise, real-time and transparent spectral classification. We start by describing various statistical and machine language algorithms commonly used for LRS data processing; followed by a brief review of multiple efforts using LRS to diagnose breast cancer using these algorithms. We will point out the variety of statistical analysis and classification techniques employed in these studies with emphasis on the absence of a standard methodology for (1) spectral data collection, (2) signal pre-processing, and (3) final classification. We posit that agreement on those three steps will be critical to the creation of national database development for the use of LRS in biomolecular breast cancer diagnostics. We suspect that stochastic neural networks will become the classification strategy of choice in oncogenic diagnostics due to their ability to (1) identify complex, non-linear boundaries between classes; (2) produce probabilistic estimates of the likelihood their predictions are correct; and, (3) estimate the expected error in their own prediction. We posit that (1) the majority of so called “machine learning” algorithms are, in fact simply statistical tools; and, (2) that stochastic backpropagation algorithms are the only true learning algorithm inherently providing the two most critical pieces of information: the Bayesian probability of correct classification and an estimate of the certainty of each classification probability for each target.

## 2. Statistical and Machine Learning Methods

The preparation and use of LRS data proceeds in most studies through several steps generally summarized as (1) pre-process cleaning of the raw incoming signal; (2) identification and extraction of the portion(s) of the data containing the lion’s share of the information (dimensionality reduction); (3) the pure unsupervised preliminary clustering of the LRS target sites driven only by the extracted features without human bias; and (4) a formal classification of all targets using a gold standard for training. The gold standard can be either the output of an unsupervised clustering algorithm, or the opinion of a human expert after evaluating other data, e.g., H&E photomicrographs of the LRS targets, or, preferably, both.

**Figure 2:**
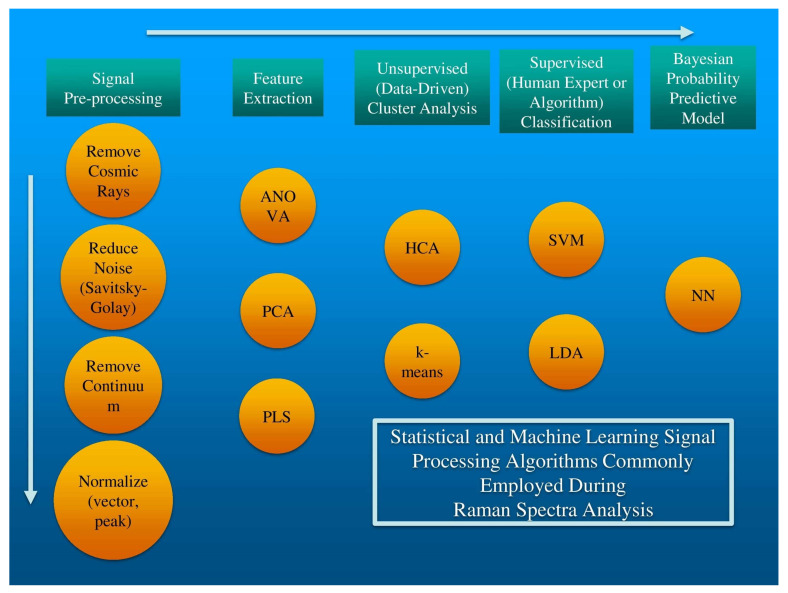
The data processing steps required for robust Raman spectral analysis with the various algorithms that can be used for each step.

Until recently, a fifth stage has often been omitted or minimized: rigorously estimating the likelihood that the putative classification decision is correct and then evaluating the replicability of that decision. We will briefly describe several algorithms commonly employed in each of the first four tasks and then describe an easily implemented stochastic neural network method to generate both a Bayesian probabilistic classification as well as error estimation for classification replicability. Figure 2 includes a flowchart of these five data processing steps as well as commonly used algorithms for each step.

### 2.1. Data Pre-Processing

Raman scattering is an example of an inelastic scattering of a photon after it interacts with the electron cloud generated by a wide variety of molecular bonds, e.g., C—H, C—H_2_, C—H_3_, —OH, etc. The change in energy is recorded in the Raman spectral data as a Stokes shift in the photon’s wavelength towards the red end of the spectrum (lower frequency, lower energy) if the electron cloud is in the ground state, and toward the blue end (higher frequency, higher energy) if the molecular bond has been excited to a higher energy state by some previous event. The molecular specificity of an LRS spectrum comes with a price. Very few laser photons striking a target will carry the vibrational energy signature of one of the molecular bonds in the target. The vast majority will be either scattered elastically without carrying vibrational information (Rayleigh scatter) or absorbed to be subsequently dissipated as heat or fluorescence.

Since the Raman effect is such a rare event, to minimize data acquisition time highly sensitive CCD and CMOS chips and thermal cooling systems are now the core sensor components for collecting Raman photons present at just above sensor thermal background levels. The sensitivities are such that an LRS spectrometer makes an excellent cosmic ray detector. Signal pre-processing starts in modern systems with a series of embedded software routines that look for spike events involving only one or two spectral bins which are characteristic of cosmic ray events [24]. Once a spike is detected, the embedded algorithm then uses any one of a number of nearest neighbor routines to interpolate and replace the affected pixels. The next step in most pre-processing protocols is usually called noise reduction or smoothing. The most common choice of algorithm for the past three decades has been that devised by Savitsky and Golay [25]. The algorithm uses a moving window based local polynomial fitting routine requiring operator decisions about parameters such the size of the moving window and polynomial order. The inherent danger in the smoothing is that as the moving window size increases, some Raman bands of interest with broad FWHM (full width half maximum) and low S/N characteristics may be lost. The third stage of preprocessing is the removal of the red-shifted fluorescence and other continuum distortions arising from variables such as changes in sample absorption characteristics as a function of wavelength, laser bleed-through, or CCD inhomogeneities. The baseline corrections that must be accomplished without distorting the information in the raw data, are quite critical, but relatively simple to automate. A wide variety of techniques have been investigated including wavelets [26], FFTs [27], first and second derivatives [28], and polynomial fitting [29]. At present the most commonly implemented baseline correction techniques involve some form of polynomial fitting [30]. An easily implemented method uses an asymmetrically reweighted penalized least squares smoothing algorithm (arPLS) developed by Baek et al. [31]. This method employs an iterative process to estimate a baseline. The calculated baseline is then subtracted from the intensity values of the raw spectrum to remove the continuum.

The final pre-processing step, normalization, attempts to minimize the impact of variables in the data collection process that are independent and extraneous to the hypothesis driving the LRS investigation [32]. Common confounding variables include changes in sample temperature, ambient lighting, laser power drift, and CCD/CMOS chip temperature. The two most common normalization procedures are peak normalization and vector normalization. In peak normalization a single peak, one predicted to be unchanging in the experimental conditions, is chosen as a gold standard. All peaks, including the normalizing peak, are then divided by the intensity of that normalizing peak. For a given set of experiments, evaluation of the entire data set using principal component analysis (PCA, defined below) can provide confirmation that the normalizing peak does not contribute to the information content of the LRS spectra. But that confirmation does not generalize beyond the bounds of the original experiment. In vector normalization, the square root of the sum of the squared intensities of the spectrum is calculated. Then, each of the Raman spectral intensities is simply divided by this ‘norm’ to obtain a normalized spectrum. Vector normalization is becoming increasingly popular and may be more useful when trying to interpret the results of disparate LRS measurements performed under a variety of experimental conditions.

### 2.2. Data Optimization and Dimension Reduction

At present it has become common practice for data analysis to begin with optimization of the raw input data using principle component analysis (PCA). PCA, also known as the Karhunen-Loeve or hotelling transform, is a classical unsupervised multivariate analysis technique. Often incorrectly thought of as a classification algorithm, PCA is actually the premier method for reducing the dimensionality of the raw data. In the case of LRS, the raw spectral data may contain ~2000 bands. PCA will often be able to reduce that enormous input vector to as few as 3–10 principal components or factors. PCA identifies linear combinations of raw parameters that account for the maximum variance. It is important to note that in machine learning and artificial intelligence, variance is often characterized as the information content in a data set. Analysis of variance (ANOVA) can be used to confirm PCA detection of high information content in specific regions of LRS spectra. The data dimensionality reduction significantly increases the computational efficiency of classification algorithms and makes it more likely that sophisticated, complex algorithms such as neural networks will not “memorize” the training set data, but will instead extract more robust correlations that can be applied to classify previously unseen data sets. Finally, PCA is unaffected by modest amounts of noise in the data since the covariance matrix is an average over many data points while the noise is uncorrelated from point to point [33].

Partial least squares (PLS) bears considerable resemblance to PCA. However, while PCA concentrates only on modeling the *y*-axis of a data set, PLS simultaneously models the structure of both the x and y axes. PLS in a form known as PLS-regression (PLSR) is used extensively in chemometrics [34]. PLSR usefulness is its ability to analyze data containing noisy, collinear, and even incomplete variables in both x and y.

### 2.3. Unsupervised, Autonomous Data Exploration

Machine learning algorithms generally fall into one of two categories: unsupervised and supervised. Unsupervised algorithms identify patterns in a data set without prior knowledge of independent data that might provide classification criteria easily interpreted by a human expert. Supervised algorithms are provided with the predicted classification of either a human expert or the results of an unsupervised algorithm. The supervised algorithm then must predict the likelihood the clustering or classification model proposed by either the unsupervised or human expert is correct.

**Figure 3:**
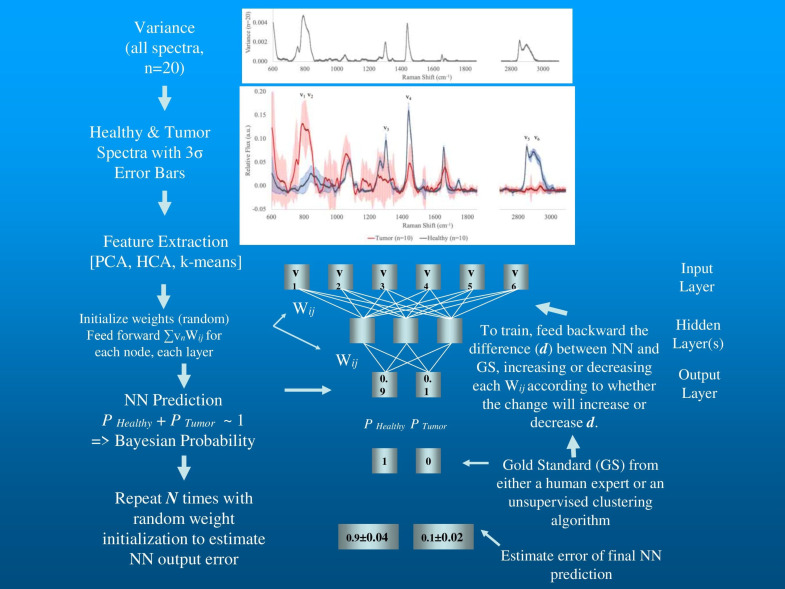
Flowchart example of data processing for LRS breast tissue signals.

When initially exploring any unknown environment, it is certainly desirable to identify self-organizing patterns in the raw data. The exercise can quickly point out the existence of anomalies in the data such as outliers, measurement results unexpected by the experiment and distant from the spectral patterns generated by all other samples. The human or algorithmic analysis of these readings is critical for proceeding with data processing since there are only two primary reasons for the existence of an outlier. The common assumption is that this is a measurement error and the offending data should be thrown out. The other possibility, of course, is that this is a serendipitous detection of an unexpected phenomenon and may be the most important finding in the experiment. Both unsupervised artificial neural networks and factor analysis techniques have been employed successfully for such an initial qualitative exploration. In the data flow illustrated in Figure 3, PCA factors extracted from raw data are often used as inputs for two unsupervised clustering algorithms: hierarchical cluster analysis (HCA) and k-means. HCA is a completely autonomous cluster detection algorithm used extensively in molecular biology, medicine, and biochemistry to explore genome data sets [33,35].

Dendritic tree HCA initially considers each member of the data set as a separate cluster and then combines the clusters by merging nearest neighbors. Repeated with the resulting combined data points the binary combination continues, reducing the number of clusters at each step, until only one cluster remains. The HCA data partitioning is then represented by a dendrogram, or a tree where each step in the clustering process is illustrated by a node or branching of the tree. The distance between each level is calculated, and significance levels can be set deciding if the data best described by 1, 2, 3,..., N clusters. HCA can be used as the very first data exploration algorithm since it does not require any help from a human expert, not even a priori selection of the probable number of classes. The algorithm can also generate a corresponding tree diagram showing contribution of each member of the input vector to the final clustering partitions. Such a dendritic tree produced the canonical genomic classification system devised by Woese et al. [36], a central tool for understanding the diversity and evolution of life.

The second unsupervised algorithm, k-means, has been shown to be able to generate the optimal clustering model for any input data [37]. However, it is not quite as autonomous as HCA. K-means requires an initial estimate of the likely number of clusters if it is going to solve the clustering task in a reasonable amount of time. The estimate of the likely number of clusters can be provided automatically as one of the outputs for HCA, or it can be provided by a human expert. After choosing the initial data points as temporary cluster centers, k-means assigns data points to a cluster and moves the center of the cluster such that the sum of the squared distance between the data points and the arithmetic mean of all the cluster’s data points is minimized. The algorithm does not generate a probabilistic estimate of the accuracy of cluster assignment.

### 2.4. Supervised Data Classification

Linear discriminant analysis (LDA) and support vector machines (SVM) are supervised machine learning classifiers. Often referred to as Fisher’s linear discriminant, LDA, like PCA, searches for a linear combination of variables that optimally characterizes a data set [38]. Unlike PCA, LDA has access to not only the uncontrolled variables (in the case of LRS the spectral bands), but also to the predicted classification of, usually, a human expert. Given that a priori classification, LDA attempts to model the differences between the predicted classes.

SVM is a robust, easily implemented and reliable linear binary classifier. SVM can rapidly identify class boundaries between two linearly separable clusters [39]. Vectors of the input data are non-linearly mapped to a high-dimensional feature space where a linear decision surface is formed to create two clusters [40]. Kernel trick is often employed when using SVM so that one can operate in input space instead of a highly dimensional one [41]. SVM boundary decisions are relatively easy to understand. However, the final classification is purely binary. Neither LDA or SVM produce a probability that a classification is correct and do not inherently estimate the error in their prediction for each target.

### 2.5. Bayesian Probabilities of Correct Classification—Stochastic Neural Networks

Once unsupervised algorithms assign cluster memberships to each data point for all samples, the correctness of each assignment must be estimated. Bayesian probability theory is a powerful formalized method to generate the probability that each data point should be included in each of the data clusters proposed by either a human expert or an unsupervised algorithm [42]. Stochastic backpropagation neural networks (NNs) are simple optimization algorithms modeled on the neuronal signal processing characteristics of the brain [43,44,45,46,47]. These stochastic algorithms have been shown to be robust estimators of the Bayesian a posteriori probability of correct classification and easily generate an error analysis for each probability [48,49]. Unlike LDA and SVM, NNs can model non-linear boundaries, have been shown to be universal approximation machines, and can generate a Bayesian probability that each target has been correctly classified [50]. NNs are not confined to binary classification tasks, but can easily assign a Bayesian probability that a target is a member of each class under consideration. Breast cancer tissue is often heterogenous [51], and at a given time the Raman laser spot light might be incident on a boundary region and thus capturing signal from a mixture of malignant and healthy cells. Hence a probabilistic classification model can more accurately depict the heterogenous nature of cancer and leave the final decision making to the surgeon.

Backpropagation networks consist of two or more “layers of nodes”. A node (***i***) is just one of the numbers in a vector describing a data point. In NN terminology, a complete vector is called a layer (***j***). Each NN will have at least one input layer of nodes, an output layer, and a set of weights (***w_ij_***) that fully connect the nodes of the two layers. The output nodes for the NN are the Bayesian probability that a target has been correctly classified either by HCA, k-means, or a human expert. If the classes are linearly separable, only the input and output layers and their connecting weights are required. If a nonlinear separation is expected, one or more layers are inserted between the input and output layers to extract higher order terms.

Figure 3 illustrates a simple data flow we have used in previous work beginning with the initial analysis of variance and choice of spectral regions of interest used as qualitative input exploration with PCA, HCA, and k-means, through to a stochastic backpropagation NN using a single intermediary layer between the input and output layers (known as the hidden layer) [52].

For this strawman example Figure 3 depicts the variance in 20 LRS spectra acquired while evaluating ex vivo tissue excised during breast conserving lumpectomy, 10 from sites identified by subsequent histopathology as tumor sites and 10 from sites identified as healthy tissue. The spectra have been corrected for cosmic rays, smoothed using Savitsky-Golay algorithm, continuum removed by arPLS, and vector normalized. The first frame depicts the variance present in all 20 spectra without attention to probable classification. Multiple spectral regions appear to contain significant information. The second frame depicting the 3-sigma error for spectra from each of the two putative classes, tumor and healthy, show considerable overlap for several of the possible bands of interest for classification. Such a qualitative assessment can then be verified using PCA to look for spectral regions that can contribute to classification. HCA and k-means unsupervised classifications using multiple combinations of possible bands of interest can also be used to explore for the optimal data dimension reduction and clustering partitions. In the mock-up depicted in Figure 3, six regions of interest show high variance and are free from overlap at the 3-sigma level. The Raman shift values and the molecular targets exhibiting Raman shifts at these wavelengths appear in Table 1.

The intensities of each of the bands representing the spectral regions of interest enter the NN at the input layer. Each node in the hidden layer is connected to each node in both the input and output layers by a weight that is initialized at the start of training with a random number between 1 and 0. Training is a simple matter of iteratively adjusting each of these weights to generate values at the output layer that match values presented as the predicted classification. For example, a node at layer ***j*** initiates a calculation of the sum of the inputs from the previous layer each multiplied by the specific ***w_ij_*** for that connection. The node constrains its output by transforming the sum using a nonlinear threshold function of sigmoid form across the interval [0, 1]. The weights are modified by minimizing least squares (see Storrie-Lombardi et al. [49] for a detailed description of NN training). The novel aspect of stochastic backpropagation NNs is the manner in which the optimization is performed using the chain rule (also known as gradient descent), which was independently discovered by several investigators [45,54,55]. During training, the algorithm measures the differences between its output and the putative class assignment and then propagates error backward by modifying the weights in each layer according to their contribution to the total error. Note that the NNs represented in Figure 3 are non-linear, unlike the two unsupervised clustering algorithms. These NNs are capable of drawing more complex class boundaries than can be accomplished by the two clustering algorithms. Interestingly, if a target has been incorrectly assigned by one of the linear unsupervised algorithms or a human expert, these stochastic non-linear NNs will “refuse” the putative assignment of the target and generate a low probability that the assignment is accurate. In analyzing the output of these NNs, if the model has been correctly configured, the probabilistic predictions for all classes considered will sum to ~1, the required hallmark of a Bayesian analytical engine.

For data sets of limited size, training and testing of all samples can be easily accomplished by two different techniques: leave-one-out cross-validation and jackknife training/testing sequence. Both have their advantages and disadvantages. Leave-one-out cross-validation removes one target before training begins, trains an NN on the full data set less one, tests the hold out target, and then repeats the entire process for the next datum until all targets have been tested. The process is excellent for optimal utilization of small data sets, for the proper training of the NN, and for the precise evaluation of each target. But for large data sets the technique is computationally resource-intensive. A variety of jackknife procedures have been developed that generally involve selection of random subsets of the data for training, testing, and validation. It is the stochastic reset of the NN’s weights for multiple runs that makes it possible to implement a Monte Carlo test-retest sequence and produce statistics that the NNs use to generate a true error estimate to assess their own performance. It should be noted that larger data sets (about an order of magnitude more spectra than the number of weights in the NN) are preferred since their size inhibits the NNs from “memorizing” the “correct” answers given by the unsupervised algorithms or the human expert. Instead the algorithms will learn the more robust patterns in the correlations and anti-correlations driving the unsupervised clustering algorithms.

Finally, it cannot be overemphasized that since these are stochastic NNs, the entire training and testing process can be automatically run as many times as necessary with a complete random reset of the starting weights occurring at the beginning of each run. The output from these runs, that each begin at unique initial states, provides clear statistics characterizing the inherent error in the Bayesian probability generated by the algorithm. This capability separates stochastic backpropagation algorithms from all other classifiers discussed here.

## 3. Review of Major Research Advancements

Table 2 summarizes all LRS breast cancer papers reviewed in this article. In 2002, Shafer-Peltier et al. [21] presented the first comprehensive and accurate morphological model of breast tissue using a Raman confocal micro imaging system by using ‘basis spectra’ of components found in breast tissue as building blocks for macroscopic samples [21]. They collected Raman micro-images of breast tissue which were used to identify nine major components as the basis spectra: cell cytoplasm, cell nucleus, collagen, fat, cholesterol-like, *β*-carotene, calcium hydroxyapatite, calcium oxalate dihydrate and water. The Raman images were correlated with phase contrast images as well as H&E stains of the same tissue section and the images were overlapped for comparison. Using linear combinations of the nine basis spectra the investigators were able to model significant Raman features of a range of breast tissues from normal to cancerous and got consistent results with the known morphology of the tissue determined from phase contrast and H&E staining. They employed the use of least squares fitting for fitting basis spectra to the Raman spectroscopic images and used PCA to identify the independently varying components. The same year Haka et al. [19] published a LRS investigation of microcalcifications found in benign and malignant breast lesions [19]. LSR probing of these microcalcifications found two distinct categories, type Ⅰ, calcium oxalate dihydrate which are benign and type Ⅱ, calcium hydroxyapatite, which can be either malignant or benign. Using PCA on the acquired spectra the investigators were able to determine that type Ⅱ microcalcifications in benign ducts have more calcium carbonate and less protein than type Ⅱ microcalcifications found in malignant ducts. They were able to discriminate microcalcifications present in benign and malignant breast ducts with sensitivity of 88% and a specificity of 93%, which is marked improvement over traditional techniques like X-ray mammograms. In a subsequent paper published in 2005, Haka et al. [18] were able to use the a linear combination of the same basis spectra introduced by Shafer-Peltier [21] to determine the contribution of each basis spectra to breast tissue specimens of normal tissue, fibrocystic lesions, fibroadenoma and infiltrating carcinoma by normalizing the fit coefficients so that they sum to 1 [18]. These were validated using H&E stains of the same tissue specimen. Further, these fit coefficients were used to make the first spectral based diagnostic algorithm to identify specific pathologies in breast tissue. This algorithm used the fit coefficients of fat and collagen to divide all data points into two categories, one group contained infiltrating carcinomas and fibroadenomas and the second group contained normal tissue and fibrocystic lesions. Logistic regression was used to further subdivide the two groups resulting in sensitivity of 94% and specificity of 96% for infiltrating carcinoma when compared to H&E analysis by the pathologist.

In 2006, the same group published their first LRS in vivo study of margins during a partial mastectomy [17]. The investigators collected a total of 31 Raman spectra from nine patients, and were able to collect the spectra in 1 s, highlighting the potential real-time benefits of this technique. The data was analyzed and fit in real-time using the same basis spectra [21] and model [18] described in previous publications by the same group. The diagnostic algorithm produced 100% sensitivity and specificity for detecting carcinoma when compared to the standard H&E pathological review; although it is important to note that there was only one malignant spectral sample in this dataset. This malignancy was grossly invisible during surgery, and after H&E analysis the margin was deemed positive and the patient had to undergo a second surgery. Had the malignant spectrum been taken into account during initial surgery, a second surgery could have been avoided altogether, hence highlighting very promising results with this first in vivo study.

Mohs et al. [56] published an in vivo study of tumor detection in mouse models in 2010 [56]. They investigated the use of a hand-held Raman spectroscopic device operating at 785 nm, called SpectroPen to detect intraoperative contrast agents. They discussed the design and performance of the SpectroPen, which can resolve NIR fluorescence from the Raman signal using optical filtering fitted into the hand-held device and compared the results from the SpectroPen to a classic 785 nm Raman spectrometer. They found that the SpectroPen was able to detect two contrast agents which adhere to malignant cells, fluorescent contrast agent (indocyanine green, ICG) and a surface-enhanced Raman scattering (SERS) contrast agent (pegylated colloidal gold). Furthermore, they studied the performance of the SpectroPen in determining tumor margins of mice injected with 4T1 tumor cells and the ICG contrast agent. They collected 14 spectra using SpectroPen and reliably found ICG signals which were correlated to the bioluminescent and bright-field images of the mouse, thus accurately detecting tumor borders.

Keller et al. [57] propose the use of spatially offset Raman spectroscopy (SORS) for tumor detection [57,67]. SORS is a technique in which Raman spectra is collected from regions spatially offset by varying amounts from the point of original incidence, which facilitates signal collection from deeper layers, since Raman photons from deeper within the sample are shifted laterally before emission from sample surface [68]. This can be instrumental in accurately determining tumor margins since clear margin is 2 mm for infiltrating carcinoma [4]. Keller et al. [57] discuss the development of a SORS probe including orientation of source and collection fibers and collected in vitro breast tissue signals [57]. The tumor signals were characterized by a strong phenylalanine band at 1006 cm^−1^ and a wider amide Ⅰ band at 1656 cm^−1^, the healthy signals exhibited a more intense CH stretch at 1445 cm^−1^ and the presence of a carbonyl stretch band at 1750 cm^−1^ and both types of signals had significantly different ratios of 1303 to 1265 cm^−1^ bands which is indicative of the ratio of lipid to protein content. They achieved a sensitivity of 95% and specificity of 100% using sparse multinomial logistic regression when compared to H&E histology.

Brozek-Pluska et al. [58] present another study of LRS data from ex vivo breast tissue with 321 spectra from 44 patients [58]. They found that normal breast tissue spectra exhibited C–C, and C=C stretching bands of carotenoids as well as the C–H symmetric and asymmetric bands of lipids, which were absent in malignant and benign tissue spectra. Additionally, their results showed more autofluorescence in malignant tissue than in normal and benign tissue and showcased a sensitivity of 72% for identifying malignant tissue using PCA analysis (using PC1). A subsequent paper published by Abramczyk et al. [59] from the same laboratory with 1100 spectra from 99 patients confirmed all of Brozek-Ploskas’ results; also obtaining a sensitivity of 72% for identifying malignant tissue with respect to the first PC in PCA analysis [59].

Kong et al. [69] published a seminal study combining auto fluorescence (AF) images to highlight important sampling points for Raman spectroscopy to diagnose basal cell carcinoma during Mohs surgery, and thereby reducing acquisition time for Raman spectra [69]. They found that this methodology was faster than frozen section histology and studies that use only infrared or Raman microscopy. PCA and K-means clustering were employed to achieve 100% sensitivity and 92% specificity. Shipp et al. [60] recently extended this methodology to BCS. The investigators used a diagnosis model and an initial independent test based on smaller mastectomy samples with sensitivity = 91% and specificity = 83% and validated data using k-means and LDA. They analyzed 51 fresh BCS specimens with multi-modal spectral histopathology (AF and Raman spectra) and could detect residual and small tumors on the surface of whole BCS samples with 100% sensitivity and at least 80% specificity.

Garcia-Flores et al. [61] investigated breast tissue of rats using high frequency LRS on in vivo and ex vivo samples [61]. Using PCA and LDA resulted in discrimination accuracy of 77.2%, 82.3% and 100% for in vivo transcutaneous, in vivo skin-removed and ex vivo spectra respectively. Zúñiga et al. [62] also employed PCA and LDA on Raman spectra of ex vivo breast tissue using a 785 nm system to get sensitivity of 90% and specificity of 86% when compared to H&E expert analysis [64].

SVM and LRS have been used to identify normal tissue, fibrocystic change (FCC), fibroadenoma (FA) and breast cancer, in the absence and presence of microcalcifications during stereotactic core needle biopsy [63]. Barman et al. [63] were able to achieve a sensitivity of 62.5% and specificity of 100% using SVM with leave-one-out cross validation. Recently LRS, PCA, LDA, and SVM have been employed to discriminate between benign lesions, fibrocystic disease, fibroadenoma, intraductal papilloma, invasive ductal carcinoma and lobular carcinoma in formalin fixed paraffin preserved tissue [64].

In the last year, a few groups have evaluated the use of neural networks for LRS breast cancer diagnosis. Shang et al. [65] used back propagation NNs on breast cancer Raman spectra [65]. They ran two separate NNs on spectra mostly comprised of collagen and those having mostly lipid content to get a discrimination accuracy of 95.33% and 98.67%, respectively. Koya et al. [66] use convolutional neural networks (CNNs) for their classification of breast tissue spectra to achieve a sensitivity of 88% and specificity of 90.8% [66].

It is important to note that menstrual status and hormonal variation can affect Raman spectra [70,71]. Kanter et al. [70] demonstrated that stratifying Raman data by a patient’s hormonal status (point in menstrual cycle and menopausal state) can increase the classification accuracy of cervical precancer from 88% to 94% [70]. In another publication, the same group found that stratifying the data by menstrual state increased the classification of low-grade squamous intraepithelial lesion (LGSIL) to 97% from 74% [71]. This finding can be extended to breast tissue as well [72,73]. Shah et al. [73] investigated the use of diffuse optical spectroscopy (DOS) to study different menopausal states in women [73]. One of their findings included higher tissue absorption coefficient before the conset of menses than before ovulation. Cubeddu et al. [72] also found that optical properties of breast tissue follow the physiological changes that occur during the menstrual cycle and these differences should be taken into account when using optical techniques on breast tissue [72].

In summary, we find that many investigators are able to produce high prediction statistics for discriminating between normal and cancerous breast tissue, but the sensitivity and specificty fall for studies that have a larger sample set. Possible compouding factors are variability in (1) menstrual state with its effect on optical properties of breast tissue, (2) percentage of lipid to fibrous tissue in healthy breast tissue, (3) tumor cell density within a healthy matrix, and, a simple item often overlooked, (4) the prescence of surgical ink used at the time of lumpectomy. Five of the six commonly used dyes that we have tested have strong contaminating Raman signals. We postulate that collecting reliable Raman spectra from all breast tissue types as well as all menstrual states that can be encountered during surgery is one way to advance the use of LRS in clinical settings. Additionally, the creation of an international database of Raman spectra of breast tissue will allow validation of diagnostic algorithms on large datasets and thus creating a more reliable, robust and accurate classification. Furthermore, augmenting Raman spectra with other imaging modalities can increase the classification accuracy and reduce data acquisition time in the operating room (as demonstrated by Shipp et al. [60]. in using AF and Raman spectra and also proposed by Maloney et al. [4]).

## 4. Conclusions

LRS efforts will continue to advance in real time surgical settings. Two series of LRS studies on brain cancer are particularly encouraging. Hollon et al. [74] have recently reported on the utility of an NN trained on over 2.5 million simulated Raman histology images to predict brain tumor diagnosis in an operating room under 150 s. They report an accuracy of 94.6% compared to pathologist interpretation which had an accuracy of 93.9% [74]. The work is an excellent example of the computational challenges amenable to current NN algorithms. Desroches et al. [75,76,77] have pioneered the operational use of a Raman spectroscopy guidance system tightly integrated with a brain biopsy needle [75,76,77]. The work points the way for LRS molecular diagnostic guidance in surgical intervention in breast, prostate, and hepatic cancer.

A fundamental LRS investigation is currently underway to understand tumor progression within a broader metabolic context. A metabolic syndrome that includes hyperlipidemia, hypertension, and diabetes is highly correlated with a decrease survival time for breast cancer patients [78]. Patients with the syndrome also exhibit a clinically covert white adipose tissue inflammation (WAT). Histologic examination of WAT reveals dead or dying adipocytes that are surrounded by macrophages [79]. Recently, LRS has been shown to be the first non-invasive technique to successfully identify the presence of WAT in both human and murine model systems [80]. The authors posit that the LRS fingerprint signal for WAT arises from fatty acid saturation that occurs in association with an adipocyte hypertrophy. The investigation opens the doorway for LRS to explore biochemical shifts in lipid pathophysiology that may be either correlated with or directly contributory to the unfolding of breast cancer tumor progression and outcome.

Clearly, over the past two decades, multiple investigators have successfully employed both PCA and analysis of variance heuristic feature selection strategies along with unsupervised and supervised clustering and classification algorithms. We would propose that it is not feature selection or choice of easily implemented binary classification tools that will decide the evolution of LRS in medical diagnostics. Instead, it will be on the LRS community to raise the bar for the use of so called “machine learning” algorithms in cancer diagnostics. We posit that the majority of machine learning algorithms employed over the last decade fail to provide the two most critical pieces of information required by the practicing surgeon: a probability that the classification of a tissue is correct, and, more importantly, the expected error in that probability. As such, they should be considered simply statistical tools to explore data sets; tools that in some ways do not qualify as learning machines. Stochastic backpropagation artificial neural networks inherently provide both pieces of information for every tissue site examined by LRS: probability of correct classification and an estimate of the reliability of that probability. If the networks are trained using both human experts and an unsupervised classification algorithm as gold standards, rapid progress can be made understanding what additional contextual data from the human expert is needed to improve classification performance.

Colleagues and patients expect us to not simply have an opinion about a putative tumor, but to know how certain we are that we our decision is correct. Only algorithms capable of providing the same two pieces of information required of the human expert should be deployed in modern LRS oncogenic diagnostics. Serendipitously, the recent wealth of work on deep neural networks (NNs with two or more hidden layers) has paved the way for mining the large, complex, multivariable data sets critical for cancer diagnostics [81,82,83]. These deep learning algorithms need to employ stochastic weight resets and standardized automatic controls for under- and over-learning during training, techniques easily implemented in even the more complex nets. We expect the coming decade to be an exciting time in the evolution of LRS and stochastic neural network technologies.

## Figures and Tables

**Table 1:** Biomolecular assignments of six regions of interest in spectra displayed in Figure 3.

Region of Interest	Raman Shift (cm^−1^)	Biomolecular Assignment * [53]
v1	780–810	O—P—O stretching DNA, ring breathing mode U, T, C bases of RNA and DNA
v2	815–825	C—C stretch of proline and hydroxyproline, out of plane ring breathing of tyrosine
v3	1302	assigned to CH2 twisting of lipids in healthy; in tumor assigned to CH2 twisting in proteins or amide III (protein)
v4	1441	CH2 bending mode in lipids
v4	2853	CH2 symmetric stretch of lipids
v4	2903	CH2 asymmetric stretch of lipids and proteins

* See Movasaghi et al. [53], and references therein.

**Table 2:** Summary of major research findings in use of LRS for breast cancer diagnostics.

Author	Tissue Type, Number of Patients, Number of Spectra	LRS System	Algorithm	Prediction Statistics	Findings
Shafer-Peltier et al. [21]	Ex vivo normal, benign and malignant tissue; For each basis spectra: 60–80 spectra from 5–6 patients; 60 Raman images	830 nm; Raman confocal microscope	Non-Negative Least squares fitting, PCA	N/A	Nine basis spectra; Raman micro spectroscopic model compared to H&E findings
Haka et al. [19]	Ex vivo microcalcifications 11 patients	830 nm	PCA	Sensitivity = 88% specificity = 93% for determining microcalcifications in malignant and benign ducts	Type ΙΙ microcalcifications in benign ducts have more calcium carbonate and less protein than type ΙΙ microcalcifications in malignant ducts
Haka et al. [18]	Ex vivo normal, fibrocystic change, fibroadenoma, and infiltrating carcinoma; 58 patients; 130 Raman spectra	830 nm	Linear combination of basis spectra, logistic regression, diagnostic algorithm based on fat and collagen content	Sensitivity = 94% specificity = 96% for infiltrating carcinoma	9 basis spectra; Fit coefficients for each basis spectra highlight chemical and morphological features of the macroscopic spectra
Haka et al. [17]	In vivo breast tissue during partial mastectomy; nine patients; 31 spectra	830 nm	Linear combination of basis spectra, logistic regression, diagnostic algorithm based on fat and collagen content	Sensitivity and specificity = 100% for carcinoma, only one malignant spectrum	In vivo spectra collected in 1 s; if malignant spectrum was taken into account during initial surgery second surgery could have been avoided
Mohs et al. [56]	In vivo mouse model; injected with 4T1 tumor cell line and ICG and SERS contrast agents; 14 in vivo spectra	SpectroPen at 785 nm	Linear regression model	N/A Validated using bright field and bioluminescence images of mouse	Descriptive development of hand-held SpectroPen compared to normal 785 LRS system
Keller et al. [57]	35 in vitro tissue samples	Spatially offset 785 nm LRS system; probe design discussed	Sparse multinomial logistic regression	Sensitivity = 95% specificity = 100% for discerning positive and negative margins	SORS allows collection of photons from deeper within the sample
Brozek-Pluska et al. [58]	Ex vivo breast tissue; 44 patients; 321 spectra	514 nm	Least squares fitting, PCA	Sensitivity = 72% for malignant tissue; sensitivity = 62% for benign tissue	Specific band and band ratio differences in malignant, normal and benign tissue discussed, malignant spectra has more autofluorescence
Abramczyk et al. [59]	Ex vivo breast tissue; 99 patients; 1100 spectra	514 nm	Least squares fitting, PCA	Sensitivity = 72% for malignant tissue; sensitivity = 62% for benign tissue; specificity = 83% for normal tissue	Same as Brozek-Pluska above
Shipp et al. [60]	51 fresh whole BCS specimens	405 nm confocal microscope for autofluoresence (AF) images, 785 nm system for Raman spectra	Unsupervised algorithm to detect segments in AF images; K-means, LDA	Sensitivity = 100% and specificity is at least 80% for multimodal spectral histopathology for 51 BCS surfaces	Results were obtained within an intraoperative timescale (12–24 min), diagnosis model trained on smaller mastectomy samples with sensitivity = 91% and specificity = 83%
Garcia-Flores et al. [61]	Ex vivo and in vivo breast tissue of rats	High-frequency (HF) Fourier transform LRS system at 1064 nm	PCA, LDA	Discrimination accuracy of 77.2%, 82.3% and 100% for in vivo transcutaneous, in vivo skin-removed and ex vivo spectra respectively	HF Raman spectra has a shorter acquisition time due to more intense signal in this region, HF region has no interfering signal from optical fiber
Zúñiga et al. [62]	Ex vivo breast tissue; six patients; 164 spectra	785 nm and 1064 commercially available systems	PCA, LDA	Sensitivity = 90% specificity = 86% with 785 nm system without microscope	Systematic comparison of 1064 and 785 nm systems with and without microscope; discussion of importance of high wavenumber signals
Barman et al. [63]	Ex vivo breast tissue; 33 patients undergoing stereotactic core needle breast biopsy procedures; 146 tumor sites	830 nm	SVM	Sensitivity = 62.5% specificity = 100%	SVM and LRS have been used to identify normal tissue, fibrocystic change (FCC), fibroadenoma (FA) and breast cancer, in the absence and presence of microcalcifications
Lyng et al. [64]	Ex vivo benign lesions (fibrocystic, fibroadenoma, intraductal papilloma) and cancer (invasive ductal carcinoma and lobular carcinoma); 20 patients	532 nm	PCA, LDA, QDA (quadratic discriminant analysis), SVM, Partial least squares discriminant analysis (PLSDA)	Sensitivity = 83% and specificity = 80% (PCA-LDA and PCA-QDA); Sensitivity = 82% and specificity = 84% (PLSDA)	Study also included immunohistochemical staining for ER and HER2 receptor
Shang et al. [65]	Ex vivo breast tissue; 14 patients	785 nm	CNN on autofluorescence images, BP-NN on Raman spectra, PLS	Discrimination accuracy of 95.33% and 98.67% respectively for collagen and lipid BP-NNs	Auto florescence images and Raman spectra fed into PLS model to achieve 100% accuracy
Koya et al. [66]	Ex vivo basal and luminal breast cancer samples	785 nm	CNN with one hidden layer	Sensitivity = 88.8% and specificity = 90.8% for discriminating cancerous and normal breast tissue	Specific band differences discussed
